# Beware of the Potential Risks for Polygoni Multiflori Caulis-Induced Liver Injury

**DOI:** 10.3389/fphar.2022.868327

**Published:** 2022-04-01

**Authors:** Wei-Song Kong, Gui Zhou, Li-Wei Xu, Kun Wang, Yi-Ming Feng, Li-Yu Tao, Rui-Fang Xie, Ming Yang, Xin Zhou

**Affiliations:** ^1^ Department of Pharmacy, Longhua Hospital, Shanghai University of Traditional Chinese Medicine, Shanghai, China; ^2^ Department of Pharmacy, Suzhou Hospital of Traditional Chinese Medicine, Nanjing University of Chinese Medicine, Suzhou, China; ^3^ Department of Pharmacy, Traditional Chinese Hospital of Lu’an, Anhui University of Chinese Medicine, Lu’an, China; ^4^ Department of Hepatology, Shuguang Hospital, Shanghai University of Traditional Chinese Medicine, Shanghai, China

**Keywords:** Polygonum multiflorum Thunb., Polygoni Multiflori Caulis, liver injury, real world, HPLC, 2,3,5,4′-tetrahydroxystilbence-2-O-β-D-glucoside

## Abstract

**Background:** *Reynoutria multiflora* (Thunb.) Moldenke (PM) is a widely-used medicinal plant in China, whose root and stem are included in the Chinese Pharmacopoeia as Polygoni Multiflori Radix (RPM), Polygoni Multiflori Radix Preparata (PMP), and Polygoni Multiflori Caulis (PMC). The hepatotoxicity of RPM and PMP is concerned by the public, while the risk of PMC is ignored.

**Purpose:** Here, we investigate the potential risks for PMC-induced liver injury from clinical, chemical, and animal features.

**Study design:** First, we analyzed the 12-month usage of RPM, PMP, and PMC in Longhua Hospital. Second, we determined the contents of gallic acid, *cis*-2,3,5,4′-tetrahydroxy-stilbene-2-O-β-D-glucoside (*cis*-SG), *trans*-2,3,5,4′-tetrahydroxy-stilbene-2-O-β-D-glucoside (*trans*-SG), emodin-8-O-β-D-glucoside (EG), physcion-8-O-β-D-glucoside (PG), emodin, and physcion in the water extracts from 15 batches of RPM, PMP, and PMC. Third, we probed the hepatotoxic effect of RPM, PMP, and PMC in mice and explored the mechanism of *cis*-SG and *trans*-SG causing the liver injury at the dosages based on our results from the first and second parts.

**Results:** PMC had nearly five times the amount of usage in both outpatient prescriptions and inpatient orders than RPM and PMP. Overall, 68% dosage of PMC was 30 g. The contents of *cis*-SG, *trans*-SG, and emodin in PMC water extracts were significantly lower than those in RPM and PMP water extracts. PMC induced milder idiosyncratic liver injury for its lower content of *cis*-SG and *trans*-SG than its root counterparts.

**Conclusion:** The potential risks for PMC-induced liver injury should be fully aware of.

## Introduction

Traditional Chinese medicine (TCM), which plays a significant role in the Chinese community, is also widely used all over the world. TCM relies on prepared slices of crude drugs which can be used for decoction or the materials for preparations. As alternative and complementary medicines, these herbal drugs are often believed to be safe but, in fact, may also induce adverse reactions like liver injury in some circumstances, which should be seriously focused (Amadi and Orisakwe, 2018; Liu et al., 2019; Zhai et al., 2021). In a retrospective study to determine the incidence and causes of drug-induced liver injury (DILI) in mainland China, TCM and dietary supplements (26.81%) were the leading single classes of implicated drugs (Shen et al., 2019). A systematic review of 9 prospective and 22 retrospective studies showed that the constituent ratio of herb-induced liver injury (HILI) among overall 7511 DILI cases was 25.0% (Byeon et al., 2019). Indeed, more emphasis should be laid on liver injury induced by those herbal medicines, for example, *Reynoutria multiflora* (Thunb.) Moldenke (synonym: *Polygonum multiflorum* Thunb., PM).

PM, also called Heshouwu in Chinese, is a popular medicinal plant based on the TCM theory (Teka et al., 2021). The Chinese Pharmacopoeia (ChP) includes two forms of root slices: raw Polygoni Multiflori Radix (RPM) and Polygoni Multiflori Radix Preparata (PMP). RPM contributes to detoxification, carbuncle elimination, and bowel relaxation, while PMP tones the liver and kidney, benefits essence and blood, blackens hair, strengthens muscles, and relieves hyperlipidemia (Liu et al., 2019). Antioxidant, antiaging, anti-inflammatory, anticancer, neuroregulatory, and hepatoprotective effects of PM were also reported by several investigations (Teka et al., 2021). In recent years however, the incidence of liver injury induced by PM and its preparations has gradually increased (Li H. et al., 2017; Wang et al., 2019). Unfortunately, the potential toxic components and possible mechanism that cause the hepatotoxicity remain in dispute. The main components in PM are stilbenes like 2,3,5,4′-tetrahydroxy-stilbene-2-O-β-D-glucoside (TSG); tannins like gallic acid; anthraquinones such as emodin, physcion, emodin-8-O-β-D-glucoside (EG), and physcion-8-O-β-D-glucoside (PG); flavonoids; phospholipids; etc. (Lin et al., 2015b; Teka et al., 2021). The current focus of hepatotoxic components has been on stilbenes and anthraquinones (Lin et al., 2015a; Xu et al., 2017; Yu et al., 2017; Xing et al., 2019). It is generally believed that processing plays a significant role in toxicity attenuation (Zhang et al., 2016; Liu et al., 2018; Liu et al., 2019). The previous reports have shown that the toxicity and the contents of the combined anthraquinones like EG are reduced after processing, indicating the combined anthraquinones are correlated with hepatotoxicity (Liu et al., 2018; Zhai et al., 2021). However, the free anthraquinones, for example, emodin, are also shown to be hepatotoxic *in vitro* and *vivo* (Liu et al., 2019). On the other hand, the findings of Li et al. reveal that *trans*-2,3,5,4′-tetrahydroxy-stilbene-2-O-β-D-glucoside (*trans*-SG), the predominant form of natural stilbene in PM, can be transformed by ultraviolet light or sunlight into *cis*-2,3,5,4′-tetrahydroxy-stilbene-2-O-β-D-glucoside (*cis*-SG) (Li C. et al., 2017). The latter isomer of TSG is responsible for the idiosyncratic hepatotoxicity in PM (Li C. et al., 2017; Meng et al., 2017).

Polygoni Multiflori Caulis (PMC) is the dried stem of PM, which is also included in ChP (Zhao et al., 2013; Wang et al., 2017; Bo et al., 2021). According to ChP, PMC is used to treat sleeping disorder, rheumatic arthralgia, and skin pruritus. Pharmacological studies have indicated its hypnosis, antidiabetic, and antioxidant effects (Bo et al., 2021). Although PMC has a larger amount of usage in clinical practice than RPM and PMP, fewer cases on liver injury induced by PMC are reported. Furthermore, there are few studies featuring the hepatotoxicity of PMC in sharp contrast with RPM and PMP. Despite different medicinal value, PMC is taken from the same plant as RPM and PMP, and the components in PMC are similar to those in RPM and PMP (Han et al., 2013; Wang et al., 2017; Rui et al., 2018; Rui et al., 2020). It can be speculated that PMC might also lead to liver injury. In this work, three interrelated parts of research are carried out to investigate the potential risks for PMC-induced liver injury from clinical, chemical, and animal features. First, we analyze the 12-month usage of RPM, PMP, and PMC in a TCM hospital. Second, we compare the chemical variations in the water extracts from 15 batches of RPM, PMP, and PMC and determine the contents of gallic acid, *cis*-SG, *trans*-SG, EG, PG, emodin, and physcion. Third, we probe the hepatotoxic effect of RPM, PMP, and PMC in mice based on the dosages in clinics analyzed in the first part and explore the major toxic ingredient and possible mechanism that cause the liver injury. The ingredient and its dosage are designed on the basis of our results in the second part.

## Materials and Methods

### Prescription, Order, and Usage Amount of Analysis

Outpatient prescriptions and inpatient orders containing RPM, PMP, and PMC in Longhua Hospital from December 1, 2016 to December 1, 2017 were chosen from the hospital information system (HIS) to analyze the information such as age, sex, and department by PA Software (self-developed, Chinese Software Copyright Registration No. 2017SR012498).

### Chemicals and Herbal Materials

Gallic acid (#B20851), *trans*-SG (#B21757), emodin (#B20240), physcion (#B20242), and EG (#B20241) were bought from Yuanye Biotech (China). *Cis*-SG (#E-0261) and PG (#E-2497) were purchased from Tauto Biotech (China). Methanol and acetonitrile (HPLC grade) were provided by Merck (Germany). Deionized water was obtained using a Milli-Q water system (Millipore, United States). Phosphate acid was from Shanghai Wujin Chemicals (China). RPM, PMP, and PMC were collected from several provinces in China (Supplementary Table S1). These samples were authenticated by Chief Pharmacist Xiu-Feng Shi from Longhua Hospital. Voucher specimens were deposited in a light-absent and well-ventilated room.

### Preparation of Herbal Water Extracts and Standard Solutions

From each batch, 25 g of RPM, PMP, and PMC samples were, respectively, boiled with 150 ml water for 1 h, filtered, then boiled with 100 ml water for 1 h, and combined together. For high-performance liquid chromatography (HPLC) analysis, the combined water extracts were concentrated to 0.5 g crude herb per mL. Then 1 ml of the concentrated extract was accurately measured, transferred to a 10-ml volumetric flask, and diluted with methanol. The mixture was sonicated for 30 min, restored overnight at 4°C, centrifuged for 15 min at 2,370 g, and filtered through a 0.22-µm microporous membrane.

Stock standard solutions of the accurately weighed reference compounds were prepared in methanol with appropriate concentrations. Then 200 μl of the standard mixture was centrifuged for 15 min at 13,680 g and filtered through a 0.22-µm microporous membrane before injection. The calibration curves were obtained by plotting the chromatographic peak areas versus the concentration of the analytes.

### Chromatographic Conditions

HPLC analysis was performed using an Agilent Technologies 1290 Infinity HPLC system (United States) coupled with a pro-shell 120 SB-C_18_ column (2.7 μm, 100 × 2.1 mm) at 30°C. The mobile phase consisted of solvent A (0.1% phosphate acid in deionized water) and solvent B (acetonitrile). The gradient elution was as follows: 5–25% B at 0–10 min, 25%–50% B at 10–15 min, 50%–70% B at 15–20 min, 70%–90% B at 20–25 min, and 90% B at 25–30 min. The flow rate was 0.3 ml/min, the injection volume was 3 μl, and the UV wavelength was set at 280 nm.

### Method Validation

The standard mixture solution was analyzed six times a day to estimate the precision. Five different sample solutions, prepared as previously described, were injected to check the repeatability. The stability test was carried out at different time points (0, 1, 2, 4, 8, 16, 20, and 24 h) with the same sample solution.

### Establishment of Fingerprints and Hierarchical Clustering Analysis

HPLC fingerprints of PM samples were established by using a similarity evaluation system for the chromatographic fingerprint of TCM (2004A), developed by the ChP Commission (China). Each established chromatographic fingerprint was recorded to present the chemical feature of RPM, PMP, and PMC. Common peaks of 15 batches of RPM, PMP, and PMC were confirmed, respectively, and merged together to compare the peak areas between RPM, PMP, and PMC in the same retention time. HCA was carried out in R to estimate chemical similarity among batches of PM based on Ward D2 linkage algorithm and Euclidean distance metric.

### Animals

Female Balb/c mice (6 weeks old) were purchased from Shanghai Model Organisms Center (China). The mice were housed at a constant temperature and humidity on a 12-h light/dark cycle with free access to food and water and were allowed to accommodate for 7 days prior to the experiments. All procedures were approved by the Animal Ethics Committee of Longhua Hospital (Ethics Approval No. SCXK2020-1061) and were performed in accordance with the guidelines of the National Animal Welfare Law of China.

The mice were divided into 12 groups (7 mice/group): control, LPS, RPM_high_, PMP_high_, PMC_high_, RPM_low_ + LPS, PMP_low_ + LPS, PMC_low_ + LPS, TSG_RPM_, TSG_RPM_ + LPS, TSG_PMP_ + LPS, and TSG_PMC_ + LPS. The procedure of herbal water extract preparation was carried out as mentioned earlier, and the extracts were concentrated to 30 g/kg for RPM_high_, PMP_high_, and PMC_high_ groups, and 6 g/kg for RPM_low_ + LPS, PMP_low_ + LPS, and PMC_low_ + LPS groups. The dosages of TSG were calculated using the ratio of *cis*-SG and *trans*-SG average contents in 30 g RPM, PMP, and PMC based on the aforementioned chemical analysis. *cis*-SG and *trans*-SG were dissolved in water and kept in light-shielded bottles. These groups were orally administrated with RPM, PMP, and PMC or TSG daily for 14 days, respectively. On day 14, the relevant groups were injected through the caudal vein with 0.5 mg/kg lipopolysaccharide (LPS, Sigma, #L2880, United States) dissolved in normal saline 2 h before oral administration, and all mice were euthanized for sample collection 6 h after gavage.

### Serum and Liver Tissue Processing

Blood samples were collected, centrifuged to obtain serum, and stored at −80°C. The serum levels of ALT and AST were determined by using a Beckman Coulter AU5800 automatic biochemical analyzer (United States). Liver tissues were placed immediately in 4% paraformaldehyde to fix overnight, dehydrated, embedded in paraffin, sliced for 5-µm-thick sections, and stained with hematoxylin–eosin (HE). For enzyme-linked immunosorbent assay (ELISA), hepatic tissues were homogenized with RIPA lysis buffer containing protease and phosphatase inhibitor cocktail (Beyotime, China). Interleukin-6 (IL-6), interleukin-1β (IL-1β), and tumor necrosis factor-α (TNF-α) in hepatic homogenates were measured by using ELISA kits (Multi Sciences (LiankeBio), China) according to the manufacturer’s protocol.

### Immunohistochemistry

Antigen retrieval and endogenous peroxidase elimination were performed in EDTA buffer (pH 9) for 5 min and in methanol containing 3% H_2_O_2_ for 10 min, respectively. The sections were then blocked in normal goat serum for 30 min, followed by incubation of primary antibodies against F4/80 (1:19200, CST, #70076, United States) and NF-κB p65 (1:400, CST, #8242, United States) at 4°C overnight and secondary antibody incubation for 30 min at room temperature. After washed with PBS, the sections were treated with DAB for 10 min, and restained with hematoxylin.

## Results

### Results of Outpatients’ Prescriptions and Inpatients’ Orders Analysis

**FIGURE 1:**
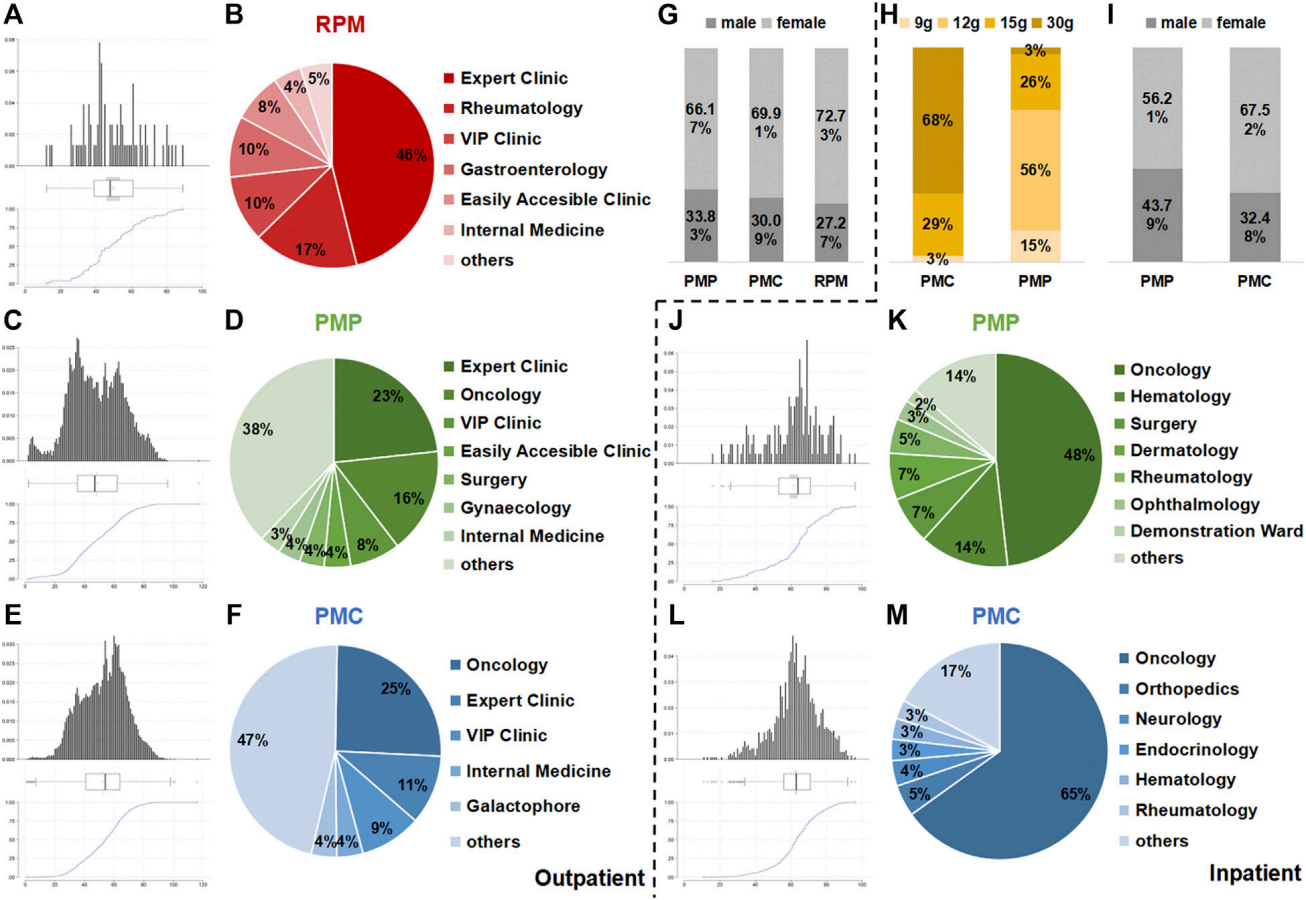
Results of outpatient prescriptions and inpatient orders analysis. Age **(A,C, and E)**, department **(B,D, and F)**, sex **(G)** of outpatient prescriptions, age **(J,L)**, department **(K,M)**, sex **(I)**, and dosage **(H)** of inpatient orders containing RPM, PMP and PMC from December 1, 2016 to December 1, 2017 in Longhua Hospital.

Of 77 outpatients, 115 prescriptions contained RPM, and 29,657 prescriptions of 12,769 outpatients contained PMP, while 1,44,025 prescriptions of 44,617 outpatients contained PMC (Table 1). Most outpatients were 40–80 years old (Figures 1A,C,E). Over 66% of outpatients using PM were female (Figure 1G). The departments prescribing PMC (Figure 1F) most frequently were oncology (25%), expert clinic (11%), and VIP clinic (9%). Expert clinic (23%), oncology (16%), and VIP clinic (8%) were the main departments prescribing PMP (Figure 1D). As for RPM (Figure 1B), the main departments were expert clinic (46%), rheumatology (17%), and VIP clinic (10%). (VIP clinic provides quality service for VIP patients. Expert clinic provides service by senior doctors.)

**TABLE 1:** Outpatient and inpatient data of PMC, RPM, and PMP.

	PMC	RPM	PMP
Outpatients	44,617	77	12,769
Outpatient prescriptions	1,44,025	115	29,657
Inpatients	1,444	—	431
Inpatient orders	4,943	—	1,109

No RPM was used in inpatient wards. In total, 1,109 orders of 431 inpatients contained PMP, while 4,943 orders of 1,444 outpatients contained PMC (Table 1). Most inpatients were 50–80 years old (Figures 1J,L). Over 55% of inpatients using PM were female (Figure 1I). The departments prescribing PMC (Fig. 1M) and PMP (Figure 1K) most frequently were both oncology (65 and 48%). The dosages of PMC were 9 g (3%), 15 g (29%), and 30 g (68%), while the dosage ChP recommended was 9–15 g. As for PMP, 12 g (56%) was the most frequently used dosage, and 15 g accounted for 15% and 30 g was 3%, compared with the dosage recommended by ChP: 6–12 g (Figure 1H).

### Method Validation Results

A good linearity of each marker ingredient was observed in a relatively wide concentration with a correlation coefficient above 0.999 (Supplementary Table S2). The relative standard deviation (RSD) of the relative peak area was calculated to verify the precision, repeatability, and stability of the method with 7 marked ingredients as the reference. Statistical analysis showed that the RSDs were lower than 1.19, 5.00, and 4.11%, respectively (Supplementary Table S2). All these demonstrated that the proposed methods were reliable for sample determination.

### Comparison of PMC, RPM, and PMP in Chemical Characteristics

**FIGURE 2:**
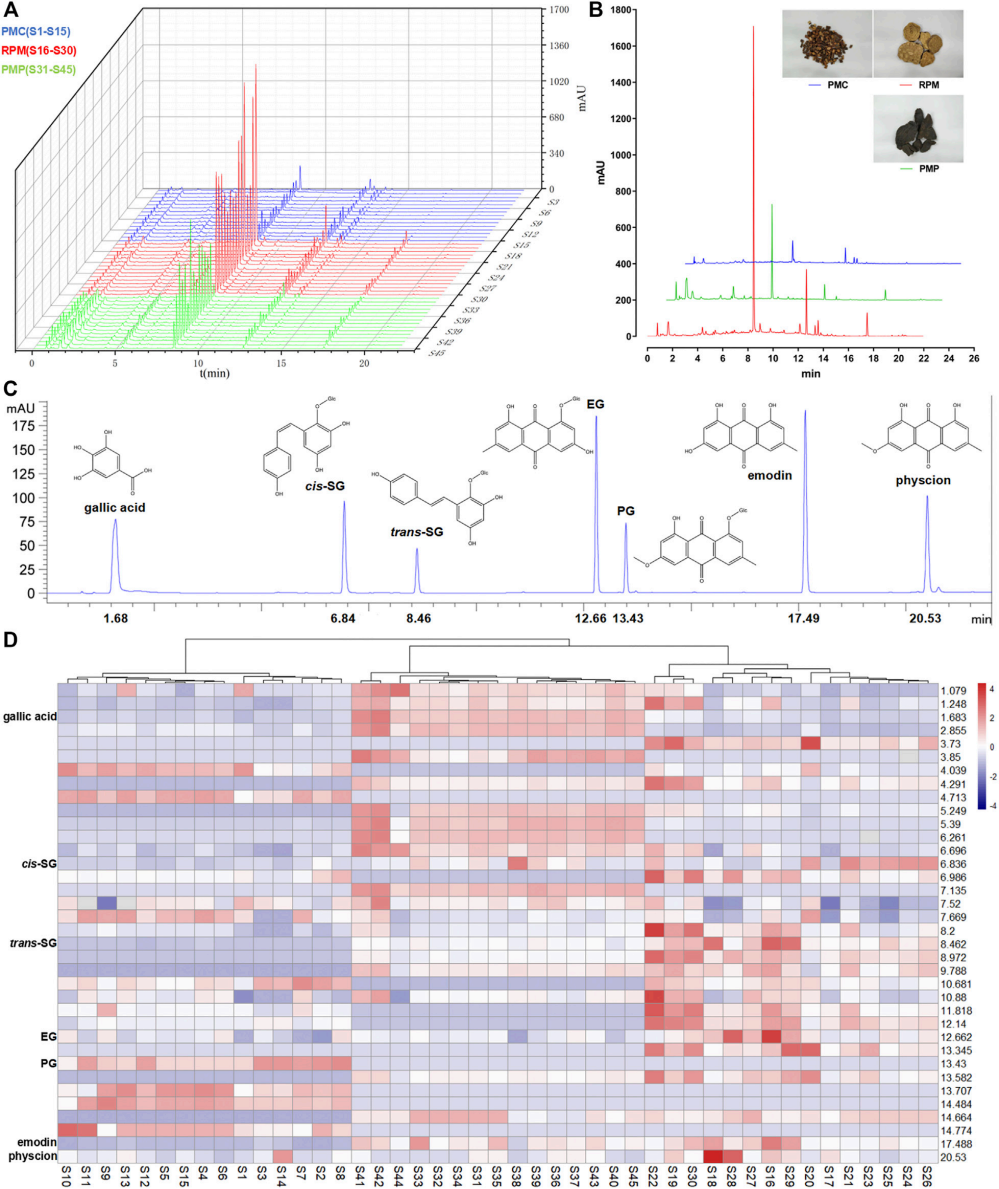
Comparison of PMC, RPM, and PMP in chemical characteristics. **(A)** Chromatograms for water extracts from 15 batches of PMC, RPM, and PMP. **(B)** Comparison of the established fingerprints. **(C)** Chromatogram of a mixture of reference compounds. **(D)** HCA heat-map for 15 batches of PMC, RPM, and PMP. Each colored cell corresponded to a peak area, with samples in rows and retention times in columns.

Fifteen batches of PMC, RPM, and PMP water extracts were analyzed by HPLC/UV under the aforementioned chromatographic conditions, and their chromatograms were recorded (Figure 2A). Thirty-six common peaks were observed between 1 and 22 min time intervals in all batches. Among them, 7 common peaks were identified as gallic acid, *cis*-SG, *trans*-SG, EG, PG, emodin, and physcion by comparing with the peaks of mark ingredients (Figure 2C). In general, the peaks in the established PMC fingerprint were lower than those in the established PMP and RPM fingerprints (Figure 2B). The peak of *trans*-SG in the established RPM fingerprint was the highest peak among all established fingerprints. HCA results were displayed on the edge of heat-map showing that the samples were properly sorted into three main clusters (Figure 2D). The left cluster consisted of S1-S15, the right cluster consisted of S16-S30, and the cluster in the middle consisted of S31-S45, corresponding the samples of PMC, RPM, and PMP, respectively. The middle and the right clusters were linked together, revealing that PMP was more similar to RPM than to PMC. In the heat-map, the blue–white–red color gradient indicated the peak area in each retention time from lowest to highest intensity. The heat-map inferred that roughly, with the retention time increase, the chemical constituents were successively concentrated in PMP, RPM, and PMC.

### Comparison of Mark Ingredients’ Contents

**FIGURE 3:**
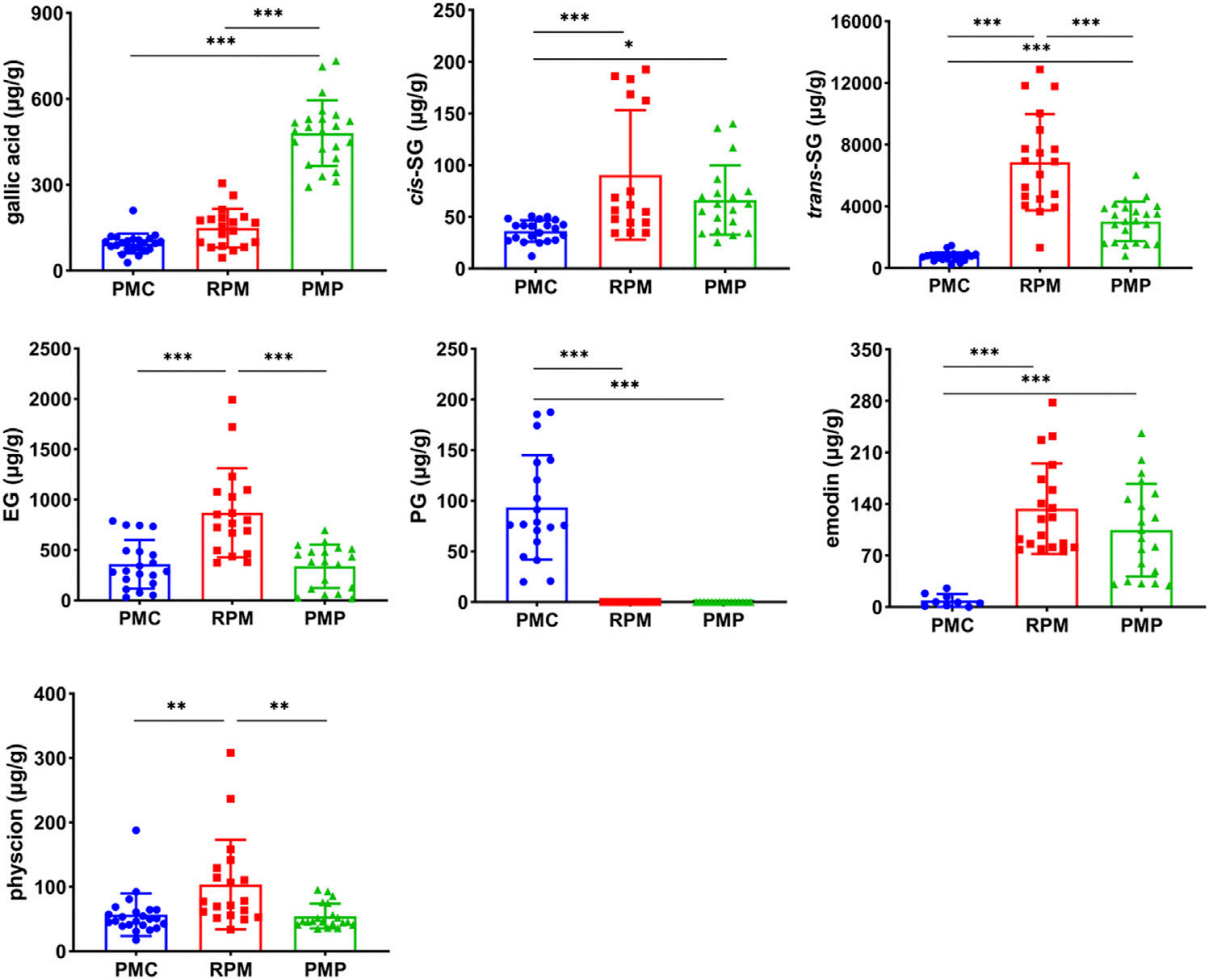
Contents comparison of gallic acid, *cis*-SG, *trans*-SG, EG, PG, emodin, and physcion in PMC, RPM, and PMP. Mean ± SD, *p < 0.05, **p < 0.01, ***p < 0.001 by ANOVA.

The contents of gallic acid were abundant in PMP, and the contents of PG were abundant in PMC, while the contents of *trans*-SG, EG, and physcion were abundant in RPM. In addition, the contents of *cis*-SG, *trans*-SG, and emodin in PMC were statistically lower than those in RPM and PMP. As for the dominant chemical component in PM, *trans*-SG, the contents were significantly different (*p* < 0.001), decreasing in the order of RPM, PMP, and PMC (Figure 3).

### Development of PM- and TSG-Induced Liver Injury in LPS-Injected Mice

The dosages of TSG were calculated according to the ratio of *cis*-SG and *trans*-SG average contents in 30 g RPM, PMP, and PMC based on the results of content determination (Figure 3). Therein, 0.462 mg/kg *cis*-SG and 40 mg/kg *trans*-SG were administrated to TSG_RPM_ and TSG_RPM_ + LPS groups, 0.45 mg/kg *cis*-SG and 18 mg/kg *trans*-SG were administrated to the TSG_PMP_ + LPS group, and 0.18 mg/kg *cis*-SG and 4.8 mg/kg *trans*-SG were administrated to the TSG_PMC_ + LPS group.

**FIGURE 4:**
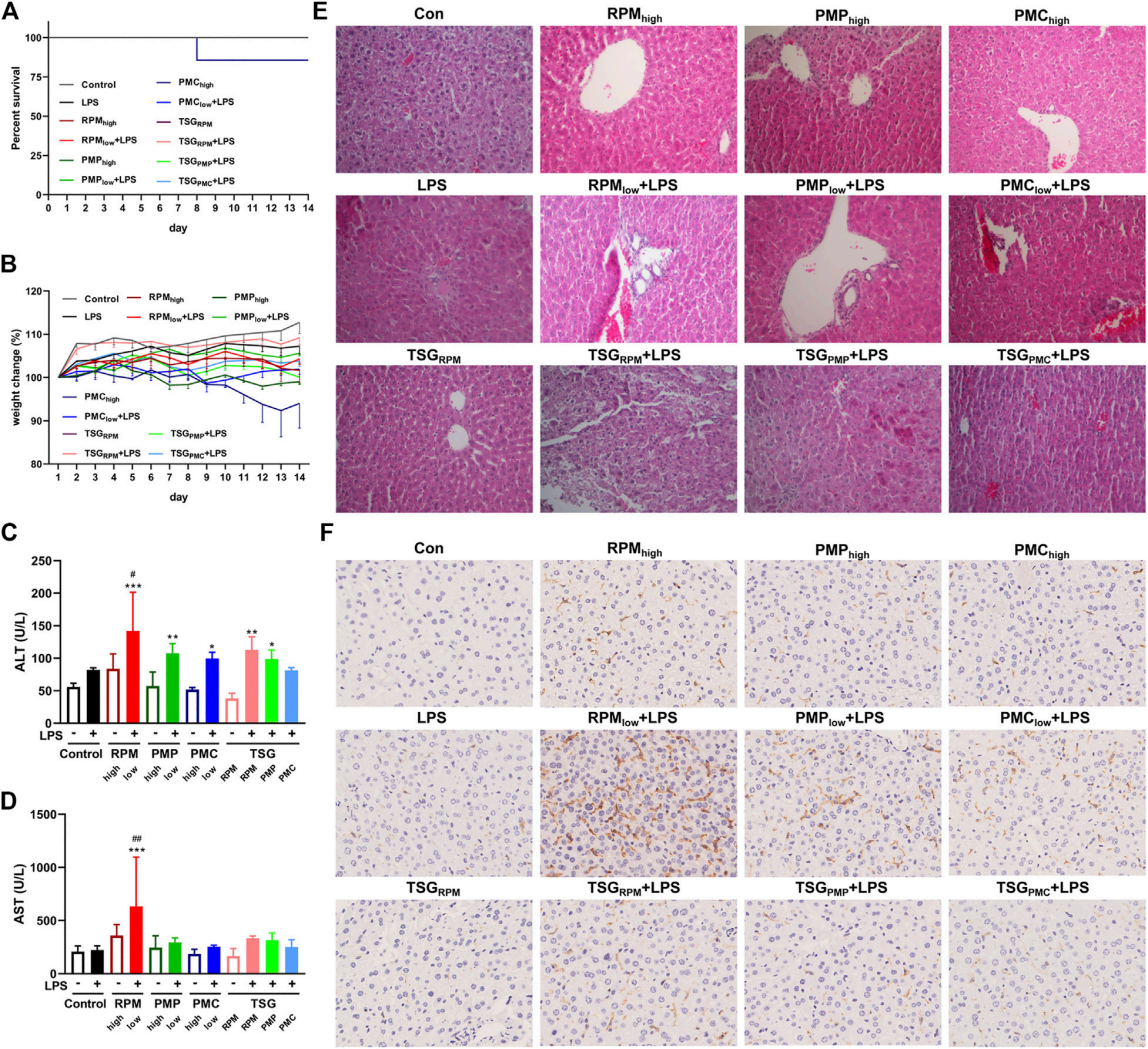
Development of PM- and TSG-induced liver injury in LPS-injected mice. RPM_high_, PMP_high_, PMC_high_: 30 g/kg/d, 14 days, ig. RPM_low_, PMP_low_, PMC_low_: 6 g/kg/d, 14 days, ig. TSG_RPM_: 0.462 mg/kg/d *cis*-SG and 40 mg/kg/d *trans*-SG, 14 days, ig. TSG_PMP_: 0.45 mg/kg/d *cis*-SG and 18 mg/kg/d *trans*-SG, 14 days, ig. TSG_PMC_: 0.18 mg/kg/d *cis*-SG and 4.8 mg/kg/d *trans*-SG, 14 days, ig. LPS: 0.5 mg/kg, 2 h before ig on day 14, iv. All mice were killed for sample collection 6 h after ig on day 14. **(A)** Survival percent of each group during the treatment (*n* = 7). **(B)** Body weight changes of each group during the treatment (n = 6, mean ± SEM). The serum levels of ALT **(C)** and AST **(D)** of each group (*n* = 6). Mean ± SD, *p < 0.05, **p < 0.01, ***p < 0.001 vs. control group, #*p* < 0.05, ##*p* < 0.01 vs. LPS group by ANOVA. **(E)** Representative images of HE staining from liver sections of each group (200×). **(F)** Representative IHC images of F4/80 staining from liver sections of each group (400×).

Poor appetite and lethargy were observed in the PMC_high_ group, and one of them began losing weight since day 4, so we suspended its PMC gavage and gave remedy since day 6 for animal welfare considerations, but it still died on day 8 (Figure 4A). One of the remaining mice in the PMC_high_ group (n = 6) began losing weight since day 11 (Figure 4B). No death or weight loss was recorded in other groups (Figures 4A,B). There were no significant differences in the levels of serum ALT and AST between the control group and LPS group, indicating the mice were injected with LPS at a non-toxic dosage (Figures 4C,D). The ALT levels of RPM_low_ + LPS, PMP_low_ + LPS, PMC_low_ + LPS, TSG_RPM_ + LPS, and TSG_PMP_ + LPS groups significantly increased compared with the control group (Figure 4C), and the AST level of the RPM_low_ + LPS group significantly increased compared with the control group (Figure 4D). HE results showed that inflammatory lesions increased in the liver tissues of RPM_low_ + LPS group, while RPM_high_, PMP_high_, and PMC_high_ groups exhibited similar liver histology to that of the control group (Figure 4E). Macrophage is one of the effector cells involved in hepatic injury (Meng et al., 2017). Herein, we investigated the expressions of macrophage-specific marker F4/80 in the liver tissues of mice. IHC results illustrated that the F4/80 expressions of LPS and TSG_RPM_ groups did not change obviously compared with the control group, while the F4/80 expressions dramatically increased in the RPM_low_ + LPS group and slightly increased in RPM_high_, PMP_high_, PMC_high_, PMP_low_ + LPS, PMC_low_ + LPS, TSG_RPM_ + LPS, TSG_PMP_ + LPS, and TSG_PMC_ + LPS groups (Figure 4F).

### PM- and TSG-Induced Liver Injury Developed in LPS-Injected Mice *via* NF-κB Signaling

**FIGURE 5:**
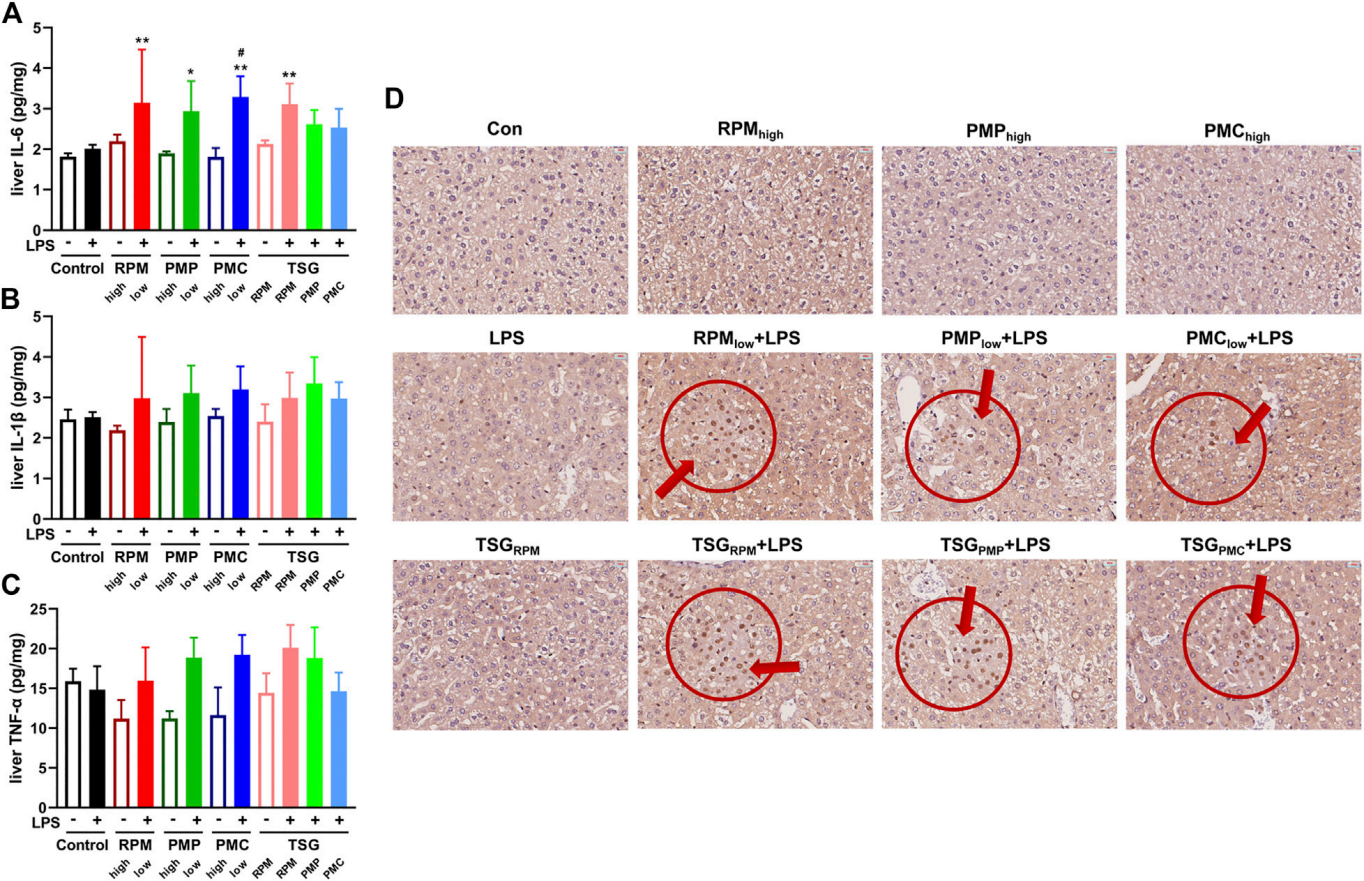
PM- and TSG-induced liver injury develop in LPS-injected mice via NF-κB signaling. RPM_high_, PMP_high_, PMC_high_: 30 g/kg/d, 14 days, ig. RPM_low_, PMP_low_, PMC_low_: 6 g/kg/d, 14 days, ig. TSG_RPM_: 0.462 mg/kg/d *cis*-SG and 40 mg/kg/d *trans*-SG, 14 days, ig. TSG_PMP_: 0.45 mg/kg/d *cis*-SG and 18 mg/kg/d *trans*-SG, 14 days, ig. TSG_PMC_: 0.18 mg/kg/d *cis*-SG and 4.8 mg/kg/d *trans*-SG, 14 days, ig. LPS: 0.5 mg/kg, 2 h before ig on day 14, iv. All mice were euthanized for sample collection 6 h after ig on day 14. The contents of IL-6 **(A)**, IL-1β **(B)**, and TNF-α **(C)** in the hepatic homogenates of each group by ELISA (*n* = 6). Mean ± SD, *p < 0.05, **p < 0.01 vs. control group, #*p* < 0.05 vs. LPS group by ANOVA. **(D)** Representative IHC images of NF-κB p65 staining from liver sections of each group (400×).

NF-κB signaling plays a critical role in inflammatory response which constitutes one of the main features of DILI (He et al., 2017; Giridharan and Srinivasan, 2018). Herein, we investigated the expressions of NF-κB p65 in the liver sections and the contents of inflammatory cytokines, IL-6, IL-1β, and TNF-α, in the liver homogenates of mice by IHC and ELISA. Compared with the control group, the contents of hepatic IL-6 were significantly higher in RPM_low_ + LPS, PMP_low_ + LPS, PMC_low_ + LPS, and TSG_RPM_ + LPS groups (Figure 5A). As for the contents of hepatic IL-1β and TNF-α, uptrends were found in the LPS-injected mice co-treated with PM or TSG when compared with the mice administrated with PM or TSG alone (Figures 5B,C). IHC results demonstrated that the expressions of p65 increased in RPM_low_ + LPS, PMP_low_ + LPS, PMC_low_ + LPS, TSG_RPM_ + LPS, TSG_PMP_ + LPS, and TSG_PMC_ + LPS groups, while the expressions of p65 in LPS, TSG_RPM_, RPM_high_, PMP_high_, and PMC_high_ groups were not obviously altered compared with the control group (Figure 5D).

## Discussion

Despite originating from the same species, PMC faces a different attitude by the public from its root counterparts. It is generally believed that PMC is much safer than RPM, given that PMC exhibits totally different efficacy from RPM or PMP according to the TCM theory (Yeung et al., 2012; Teka et al., 2021). However, a few severe liver injury cases related to TCM decoction and TCM preparation containing PMC have been reported recently in China (Zhang et al., 2019; Gao and Tan, 2020). Herein, we analyzed the outpatient and inpatient usage of PMC, RPM, and PMP through HIS from December 1, 2016 to December 1, 2017 in Longhua Hospital. PMC, as indicated in our data, has nearly five times the amount of usage in both outpatient prescriptions and inpatient orders than that of RPM and PMP (Table 1). Besides, nearly 70% dosage of PMC is 30 g, which is above the dosage ChP recommended, in contrast to the caution use of RPM and PMP (Figure 1H). The circumstances that PMC is more widely used in clinical practice with high dosage impel us to focus on the potential risks for PMC-induced liver injury.

We then compare the chemical variations among RPM, PMP, and PMC. The previous studies revealed that the contents of stilbenes, especially TSG, were higher in RPM than in PMC (Wang et al., 2017; Rui et al., 2018; Rui et al., 2020). This study also shows that the contents of gallic acid, *cis*-SG, *trans*-SG, EG, emodin, and physcion are lower in PMC than in RPM and PMP (Figure 3). It is noteworthy that the previous studies (Wang et al., 2017; Rui et al., 2018; Rui et al., 2020) investigated the powders of PM, while in this study, we prepare the water extracts of PMC, RPM, and PMP since water decoction is the major administration form of TCM in clinics (Han et al., 2013). These differences in chemical features are like two sides of a coin. The low contents of the constituents not only render PMC low risks of hepatotoxicity but also result in totally different efficacy from RPM or PMP because the potential hepatotoxic chemicals in PM are meanwhile the active ingredients (Li H. et al., 2017; Ruan et al., 2019).

We proceed our investigation on the hepatotoxic effect of RPM, PMP, and PMC in an animal model. It is reported that 75.6 g/kg (equal to 840 g/d for human) of RPM 50% alcohol extracts did not induce obvious liver injury in normal rats, whereas co-treatment with RPM extracts at 1.08 g/kg (equal to 12 g/d for human) and LPS at a non-toxic dose led to liver injury in a rat model, suggesting that PM-induced liver injury was idiosyncratic (Li et al., 2015; Rao et al., 2021; Zhai et al., 2021). Our data partly support the previous findings. The levels of ALT and AST do not change obviously in mice when treated with the water extracts of RPM, PMP, or PMC alone at 30 g/kg (equal to 150 g/d for human) for 14 days. Nevertheless, LPS combined with RPM water extracts at 6 g/kg (equal to 30 g/d for human) not only increases the levels of ALT and AST in serum significantly but also dramatically increases F4/80 macrophage expression in the liver sections of mice (Figure 4). The results are similar when we evaluate the contents of IL-6, IL-1β, and TNF-α in the hepatic homogenates and the p65 expressions in the liver sections (Figure 5). In addition, it is generally believed that the toxicity of RPM can be attenuated by processing (Zhang et al., 2016; Liu et al., 2018; Liu et al., 2019). In this work, we find mild liver damage in the PMP_low_ + LPS group compared with severe liver damage in the RPM_low_ + LPS group. As for the PMC_low_ + LPS group, the F4/80 macrophage expression slightly increased in the hepatic sections, while the serum ALT and AST do not increase obviously, indicating slight liver injury (Figure 4). Notably, poor appetite, lethargy, weight loss, and death are recorded in the PMC_high_ group (Figures 4A,B), but we cannot conclude these phenomena are interrelated to the liver injury since our results of liver biochemistries, HE staining, ELISA, and IHC do not suggest the occurrence of liver damage (Figures 4, 5). Seeing that PMC is usually used for alleviating insomnia on the basis of the TCM theory (Yeung et al., 2012; Teka et al., 2021), we speculate that an overdose of PMC may result in a fatal hypnotic effect. However, more research needs carrying out to verify this speculation.

*Trans*-SG is the predominant form of natural TSG in PM, which can be transformed into *cis*-SG by ultraviolet or sunlight (Li C. et al., 2017). The previous results indicated that when combined with LPS, *cis*-SG, but not emodin or *trans*-SG, induced severe liver injury in rats at 50 mg/kg (Meng et al., 2017). Furthermore, *trans*-SG was reported to aggravate *cis*-SG-induced liver injury in the LPS-treated rat model, indicating the synergistic mechanism of *cis*-SG and *trans*-SG in the idiosyncratic PM-induced liver injury (He et al., 2017). However, the previous *cis*-SG dose is the minimum equivalent to eight times the RPM clinical dose (He et al., 2017; Meng et al., 2017). In this study, we find that 30 g is the highest dosage of either PMC or PMP (Figure 1H), which is used by inpatients in the form of TCM decoction, so we treat mice with *cis*-SG and *trans*-SG at the dosages according to the ratio of average contents in the water extracts of 30 g RPM, PMP, or PMC based on our results of content determination (Figure 3). Interestingly, our results present that the expressions of p65 in the liver sections increase in TSG_RPM_ + LPS and TSG_PMC_ + LPS groups, whereas the content of hepatic IL-6 and the serum ALT level significantly increase in the TSG_RPM_ + LPS group but not in the TSG_PMC_ + LPS group (Figures 4, 5). It can be inferred that *cis*-SG and *trans*-SG synergistically activate NF-κB signaling in the idiosyncratic PM-induced liver injury, and the idiosyncratic liver injury induced by TSG contained in PMC may be milder than that induced by TSG contained in RPM. Based on these findings, we suggest that PMC may induce milder idiosyncratic liver injury for its lower content of hepatotoxic constituent like TSG than its root counterparts.

Taken together, this study demonstrates that PMC is more liver-friendly than its root counterparts from clinical, chemical, and animal features. However, there are still risks that patients may undergo liver injury when taking PMC medication without guidance or supervision. Hence, the potential risks for PMC-induced liver injury should be fully aware of. We advise that 1) susceptible patients should use PMC with caution and 2) long-term use of PMC with high dose should be avoided. These findings lay the foundation for the follow-up research on PMC-induced liver injury.

## Data Availability

The original contributions presented in the study are included in the article/Supplementary Material, further inquiries can be directed to the corresponding author.

## Abbreviations

ChP, Chinese Pharmacopoeia; *cis*-SG, *cis*-2,3,5,4′-tetrahydroxy-stilbene-2-O-β-D-glucoside; DILI, drug-induced liver injury; EG, emodin-8-O-β-D-glucoside; HCA, hierarchical clustering analysis; HILI, herb-induced liver injury; HIS, hospital information system; HPLC, high-performance liquid chromatography; IL-6, interleukin-6; IL-1β, interleukin-1β; PG, physcion-8-O-β-D-glucoside; PM, *Reynoutria multiflora* (Thunb.) Moldenke; PMC, Polygoni Multiflori Caulis; PMP, Polygoni Multiflori Radix Preparata; RPM, Polygoni Multiflori Radix; RSD, relative standard of deviation; RUCAM, Roussel Uclaf Causality Assessment Method; TCM, traditional Chinese medicine; TNF-α, tumor necrosis factor-α; *trans*-SG, *trans*-2,3,5,4′-tetrahydroxy-stilbene-2-O-β-D-glucoside; TSG, 2,3,5,4′-tetrahydroxy-stilbene-2-O-β-D-glucoside; UV, ultraviolet.
