# Supplementary material for: Beware of the Potential Risks for Polygoni Multiflori Caulis-Induced Liver Injury
Source: Front Pharmacol. 2022 Apr 1;13:868327. doi: 10.3389/fphar.2022.868327 (PMC9010879; doi:10.3389/fphar.2022.868327)
Supplement: Supplementary file 1 [file DataSheet1.doc]

Supplementary Table 1 Summary of herbal materials

| PMC | | | RPM | | | PMP | | |
| --- | --- | --- | --- | --- | --- | --- | --- | --- |
| Sample No. | Source | Batch No. | Sample No. | Source | Batch No. | Sample No. | Source | Batch No. |
| S1 | Henan | 180624 | S16 | Guangxi | 180723 | S31 | Guangdong | 190123 |
| S2 | Guizhou | 180718 | S17 | Guizhou | 180423 | S32 | Hubei | 181104 |
| S3 | Guizhou | 180823 | S18 | Guangdong | 180528 | S33 | Guangdong | 171219 |
| S4 | Yunnan | 190729 | S19 | Guizhou | 181012 | S34 | Yunnan | 180523 |
| S5 | Hubei | 180512 | S20 | Guangxi | 181205 | S35 | Guangdong | 180917 |
| S6 | Hubei | 180518 | S21 | Henan | 180924 | S36 | Hubei | 181106 |
| S7 | Guangxi | 181103 | S22 | Yunnan | 181017 | S37 | Hubei | 181214 |
| S8 | Guangdong | 180815 | S23 | Guangdong | 180628 | S38 | Henan | 180621 |
| S9 | Henan | 180627 | S24 | Hubei | 181014 | S39 | Hubei | 180722 |
| S10 | Henan | 180606 | S25 | Guangdong | 181221 | S40 | Henan | 180522 |
| S11 | Henan | 180723 | S26 | Hubei | 181124 | S41 | Guangdong | 180922 |
| S12 | Hubei | 180319 | S27 | Hubei | 180626 | S42 | Guangxi | 180723 |
| S13 | Guizhou | 180917 | S28 | Yunnan | 180315 | S43 | Guizhou | 180325 |
| S14 | Guizhou | 181023 | S29 | Guangxi | 180724 | S44 | Guangxi | 180224 |
| S15 | Yunnan | 181112 | S30 | Guizhou | 181009 | S45 | Henan | 180622 |

Supplementary Table 2 Results of method validation of the 7 mark ingredients

|  | Calibration curve | R | Linear range (mg/mL) | Precision (n=7) | | Repeatablity (n=5) | | Stability | |
| --- | --- | --- | --- | --- | --- | --- | --- | --- | --- |
|  |  |  |  | Peak area | RSD | Peak area | RSD | Peak area | RSD |
| Gallic acid | Y=39125X+52.649 | 0.9999 | 0.0020-0.20 | 819.923±9.745 | 1.19% | 1010.023±33.156 | 3.28% | 805.75±23.721 | 2.94% |
| *Cis*-SG | Y=15822X-2.2172 | 0.9998 | 0.0060-0.024 | 412.657±2.973 | 0.72% | 34.282±1.382 | 4.03% | 41.199±0.774 | 1.88% |
| *Trans*-SG | Y=11832X+86.563 | 0.9999 | 0.0060-0.90 | 194.144±1.788 | 0.92% | 1896.046±94.881 | 5.00% | 579.232±5.681 | 0.98% |
| EG | Y=11339X+65.846 | 0.9999 | 0.0050-0.50 | 653.238±5.065 | 0.78% | 191.512±7.37 | 3.85% | 61.04±0.533 | 0.87% |
| PG | Y=19136X-5.6714 | 1 | 0.0010-0.10 | 245.755±2.055 | 0.84% | 58.795±2.141 | 3.64% | 17.566±0.175 | 0.99% |
| Emodin | Y=35385X+27.424 | 0.9999 | 0.00022-0.22 | 897.115±6.679 | 0.74% | 142.744±5.376 | 3.77% | 89.103±2.953 | 3.31% |
| Physcion | Y=5509.8X-1.8596 | 0.9999 | 0.0021-0.42 | 497.557±4.138 | 0.83% | 19.784±0.731 | 3.70% | 29.586±1.216 | 4.11% |
